# Use of medicines and other products for therapeutic purposes among children in Brazil

**DOI:** 10.1590/S1518-8787.2016050006115

**Published:** 2016-12-01

**Authors:** Tatiane da Silva Dal Pizzol, Noemia Urruth Leão Tavares, Andréa Dâmaso Bertoldi, Mareni Rocha Farias, Paulo Sergio Dourado Arrais, Luiz Roberto Ramos, Maria Auxiliadora Oliveira, Vera Lucia Luiza, Sotero Serrate Mengue

**Affiliations:** I Departamento de Produção e Controle de Medicamentos. Faculdade de Farmácia. Universidade Federal do Rio Grande do Sul. Porto Alegre, RS, Brasil; II Departamento de Farmácia. Faculdade de Ciências da Saúde. Universidade de Brasília. Brasília, DF, Brasil; III Departamento de Medicina Social. Faculdade de Medicina. Universidade Federal de Pelotas. Pelotas, RS, Brasil; IV Departamento de Ciências Farmacêuticas, Centro de Ciências da Saúde. Universidade Federal de Santa Catarina. Florianópolis, SC, Brasil; V Departamento de Farmácia. Faculdade de Farmácia, Odontologia e Enfermagem. Universidade Federal do Ceará. Fortaleza, CE, Brasil; VI Departamento de Medicina Preventiva. Escola Paulista de Medicina. Universidade Federal de São Paulo. São Paulo, SP, Brasil; VII Departamento de Política de Medicamentos e Assistência Farmacêutica - Escola Nacional de Saúde Pública Sérgio Arouca. Fundação Oswaldo Cruz. Rio de Janeiro, RJ, Brasil; VIII Programa de Pós-Graduação em Epidemiologia. Universidade Federal do Rio Grande do Sul. Porto Alegre, RS, Brasil

**Keywords:** Child, Child, Preschool, Drug Utilization, Socioeconomic Factors, Health Surveys

## Abstract

**OBJECTIVE:**

To assess the prevalence of the use of medicines and other products for therapeutic purposes in the Brazilian pediatric population and test whether demographic, socioeconomic and health factors are associated with use.

**METHODS:**

A cross-sectional population-based study (National Survey on Access, Use and Promotion of Rational Use of Medicines – PNAUM), including 7,528 children aged 12 or younger, living in urban areas in Brazil. Medicine use to treat chronic or acute diseases was reported by the primary caregiver present at the household interview. Associations between independent variables and medicine use were investigated by Poisson regression.

**RESULTS:**

The overall prevalence of medicine use was 30.7% (95%CI 28.3–33.1). The prevalence of medicine use for chronic diseases was 5.6% (95%CI 4.7–6.7) and for acute conditions, 27.1% (95%CI 24.8–29.4). The factors significantly associated with overall use were five years old or under, living in the Northeast region, having health insurance and using health services in the last 12 months (emergency visits and hospitalizations). The following were associated with drug use for chronic diseases: age ≥ 2 years, Southeast and South regions, and use of health services. For drug use in treating acute conditions, the following associated factors were identified: ≤ 5 years, North, Northeast or Midwest regions, health insurance, and one or more emergency visits. The most commonly used drugs among children under two years of age were paracetamol, ascorbic acid, and dipyrone; for children aged two years or over they were dipyrone, paracetamol, and amoxicillin.

**CONCLUSIONS:**

The use of medicine by children is considerable, especially in treating acute medical conditions. Children using drugs for chronic diseases have a different demographic profile from those using drugs for acute conditions in relation to gender, age, and geographic region.

## RESUMO

**OBJETIVO:**

Estimar a prevalência de uso de medicamentos e outros produtos com finalidade terapêutica na população pediátrica brasileira e testar se fatores demográficos, socioeconômicos e médicos estão associados ao uso.

**MÉTODOS:**

Estudo transversal de base populacional (Pesquisa Nacional sobre Acesso, Utilização e Promoção do Uso Racional de Medicamentos – PNAUM), incluindo 7.528 crianças com 12 anos ou menos de idade, residentes na zona urbana do território brasileiro. O uso de medicamentos para tratar doenças crônicas ou agudas foi referido pelo principal cuidador da criança presente na entrevista domiciliar. Associações entre as variáveis independentes e o uso de medicamentos foram investigadas por meio de regressão de Poisson.

**RESULTADOS:**

A prevalência de uso global de medicamentos foi de 30,7% (IC95% 28,3–33,1). A prevalência de uso de medicamentos para doenças crônicas foi de 5,6% (IC95% 4,7–6,7) e para condições agudas, 27,1% (IC95% 24,8–29,4). Os fatores significativamente associados com o uso global foram ter no máximo cinco anos de idade, residir na região Nordeste, ter plano de saúde e utilizar serviços de saúde nos últimos 12 meses (consultas de emergência e internações). Associaram-se ao uso de medicamentos para doenças crônicas: idade ≥ 2 anos, regiões Sudeste e Sul e utilização de serviços de saúde. Para o uso de medicamentos em condições agudas, foram identificados os seguintes fatores associados: ≤ 5 anos, Norte, Nordeste ou Centro-Oeste, plano de saúde e uma ou mais consultas de emergência. Os medicamentos com maior prevalência de uso pelas crianças menores de dois anos foram paracetamol, ácido ascórbico e dipirona; nas crianças com 2 ou mais anos, foram dipirona, paracetamol e amoxicilina.

**CONCLUSÕES:**

O uso de medicamentos na população infantil é substancial, principalmente no tratamento de condições médicas agudas. As crianças usuárias de medicamentos para doenças crônicas apresentam perfil demográfico diferente das usuárias de medicamentos para condições agudas, em relação ao sexo, à idade e à região geográfica.

## INTRODUCTION

Drug use in children differs from drug use in adults for various reasons, especially lower prevalence of chronic diseases and higher degree of uncertainty in prescription and use. The uncertainty regarding the effectiveness and safety of medicines available for this population subgroup contributes to children being considered a risk group. Most drugs used in children have only been tested in adults, and it is observed a lack of products available in age-appropriate pharmaceutical formulations and dosage forms, as well as of studies on long-term effectiveness and safety^6^.

In 2007, the World Health Organization launched the campaign “Make Medicines Child Size” and published the First List of Essential Medicines for Children, aiming to raise awareness and speed up action to improve the availability of and access to medicines that are safe and suitable for children. Regulatory measures and measures to foster the research, development, and registration of drugs for use in children have occurred in the United States (US) and the European Union. Organizations like UNITAID, Drugs for Neglected Diseases initiative (DNDi) and Medicines Patent Pool (MPP) have stimulated the development and provision of appropriate medicines for children, such as for HIV and neglected diseases^13^.

These initiatives highlight the need to develop better medicines for children, an area that is considered neglected by various international organizations. In this regard, information on the prevalence of drug use, user characteristics and medicines used by the pediatric population are important to evaluate the appropriateness of use and estimate therapeutic needs, to improve pediatric therapeutics^3^ ^,^ ^17^.

In this context, community-based epidemiological studies offer a unique opportunity to profile drug use. Such studies provide more accurate estimates and enable a greater generalization of findings compared to studies based on samples of health service users, disease-specific carriers, or on data collected for other purposes (prescriptions or dispensing data, for example)^1^.

In Brazil, no study with a representative sample of the country has been carried out, and population-based studies have been limited to samples of children living in municipalities in the South, Southeast, and Northeast Regions^8^ ^,^ ^19^ ^,^ ^20^ ^,^ ^23^. In these studies, prevalence of use of at least one medicine ranged around 50.0%, with variations according to the age group investigated.

The aim of this study was to estimate the prevalence of the use of drugs and other products for therapeutic purposes in the Brazilian pediatric population and test whether demographic, socioeconomic and health factors are associated with use.

## METHODS

The data in this analysis are part of the National Survey on Access, Use and Promotion of Rational Use of Medicines (PNAUM), a cross-sectional population-based study. The study’s population was composed of individuals aged 12 or younger living in permanent private households in urban areas within the five Brazilian regions. Specific questionnaires were answered by the primary caregiver present at the residence at the time of the interview.

The sampling plan was complex and resulted in a representative sample of the Brazilian population residing in urban areas. The composition of the sample, sampling procedures and other methodological details of PNAUM are available in a previous publication^18^.

The use of drugs to treat chronic diseases was investigated for respiratory diseases, diabetes, and other health conditions lasting six months or longer. Specific questions were formulated for chronic respiratory diseases and diabetes, since they are diseases with high prevalence among children or that require complex administration of medicines. Caregivers were asked whether the child had a medical diagnosis and indication for the use any kind of drug to treat the related chronic disease and whether such medicines was being used.

For acute conditions (occasional health problems), specific questions were asked (“In the past 15 days, did <<child’s name>> use any medicine for infection/sleeping or nervous symptoms/stomach or bowel problems/fever/pain/cold or flu/diarrhea and vomiting?”). The use of vitamins was also investigated with the question “In the last 15 days, did <<child’s name>> use any vitamin, mineral supplement, appetite stimulant or tonic?” At the end of this set of questions, the respondent was asked about the use of drugs in the previous 15 days for any other health reasons. They were asked to provide, whenever possible, the packages or prescriptions of used medicines.

Outcomes to investigate prevalence in the pediatric sample were defined as: 1) prevalence of overall drug use (use of at least one drug, regardless of reason for use); 2) prevalence of drug use for chronic diseases (use of at least one drug to treat chronic disease); 3) prevalence of drug use for acute diseases or conditions (use of at least one drug to treat acute disease or health event). The outcome variables were categorized as yes/no. Prevalence calculation included herbal medicines and other products for therapeutic purposes mentioned by respondents (dietary supplements, foods, plants, and homeopathic products).

The following demographic and socioeconomic characteristics were analyzed: gender (female; male), age (< 2 years; 2-5 years; 6-12 years), skin color (white; non-white), economic classification according to the Economic Classification Criterion developed by the Brazilian Association of Survey Companies (CCEB 2013/ABEP) (A/B, C, D/E)^a^ and Brazilian region (North, Northeast, Southeast, South, Midwest). Medical variables were: health insurance (yes; no); number of emergency visits in the last 12 months (none; one; two or more); and number of hospitalizations in the last 12 months (none; one; two or more).

The age group investigated was based on the classification adopted by the Food and Drug Administration (FDA) for clinical investigation of medicines for pediatrics^b^, more commonly used in previous studies, to facilitate comparison of results. We chose to analyze children aged 12 and under, as this is an age group in which drug use it still determined and directly supervised by parents or caregivers.

Based on the names of the products or medicines mentioned by respondents, the drugs were identified in The Brazillian Health Regulatory Agency (ANVISA) drug lists and classified according to the pharmacological agent(s) present in their composition. Drugs used in combination (containing two or more pharmacological agents in their composition) were considered just once.

In calculating prevalence of drug use, the total number of children in the weighted sample was used as the denominator, with 95% confidence intervals (95%CI). Likewise, estimates of prevalence of drug use with frequency equal to or above 0.5% also used as the denominator the total number of children present in the weighted sample, stratified by age (< 2 years; 2-5 years; 6-12 years). For the description of the most commonly used drugs, the percentage denominator was the total amount of drugs in each age group.

Pearson’s Chi-squared test was used for the bivariate comparison of percentages, considering p < 0.05 values as significant.

Associations between independent variables and outcomes were estimated by prevalence ratio (PR), with 95%CI. Poisson regression models were developed to estimate crude PR and PR adjusted for independent variables. In the first stage, independent variables were analyzed individually. Variables showing statistical significance, defined as p < 0.20 in univariate analyses were selected for the second stage, in which all variables were introduced in the multiple model. At this stage, variables showing p > 0.05 were removed from the model one by one, with “backward” selection of variables. Statistical significance of the prevalence ratios obtained in the Poisson regression models was assessed by the Wald test.

Data were stored in SPSS software for Windows version 18.0 (SPSS Inc., Chicago, IL, USA). Descriptive analyses were executed in SPSS and the Poisson regression models were executed in Stata software version 12.0 (Stata Corp LP, College Station, TX, USA), using the appropriate set of *svy* commands for the analysis of complex samples and ensuring the necessary weighting, considering the sample design.

The project was approved by the Brazilian National Commission for Research Ethics (CONEP – Opinion 398,131, September 16, 2013). Participants were only interviewed following permission by means of an informed consent signed by the researcher and the participant.

## RESULTS

The children’s characteristics are shown in Table 1. The study population is comparable to that of the 2010 Census in relation to gender (50.9% boys and 49.1% girls), age (< 2 years: 13.9%; 2-5 years: 29.0%; 6-12 years: 57.1%) and Brazilian region (North: 10.9%; Northeast: 30.7%; Southeast: 37.9%; South: 12.9%; and Midwest: 6.7%)^c^.

**Table 1:** Sample characteristics and overall prevalence of medicine use, according to demographic, socioeconomic and medical variablesa. (N = 7,528)

Variable	Sample	Overall medicine use						
	
	%	Prevalence	95%CI	p^b^	PR_crude_	95%CI	PR_adjusted_^c^	95%CI
Overall	100	30.7	28.3–33.1	-	-	-	-	-
Gender				0.27	-	-	-	-
Female	50.3	29.5	26.6–32.5		1	-	1	-
Male	49.7	31.9	28.6–35.3		1.08	0.94–1.24	1.08	0.94–1.24
Age (years)				< 0.0001				
< 2	13.5	48.0	44.8–51.2		2.01	1.77–2.29	1.78	1.58–2.00
2-5	26.9	37.1	34.4–39.9		1.56	1.36–1.78	1.33	1.17–1.52
6-12	59.6	23.8	20.9–27.0		1		1	
Skin color				0.95				
White	46.7	30.8	27.9–33.8		1	-	1	-
Non-white	53.3	30.9	28.0–33.9		1.00	0.90–1.12	1.00	0.90–1.12
Economic classification^d^				0.2				
A/B	16.8	27.0	22.9–31.4		1	-	1	-
C	56.2	31.5	28.7–34.5		1.17	0.99–1.38	1.17	0.99–1.38
D/E	27.0	31.1	27.3–35.2		1.15	0.96–1.39	1.09	0.91–1.31
Brazilian Region				< 0.0001				
North	9.9	32.5	25.5–40.3		1.24	0.94–1.63	1.22	0.97–1.53
Northeast	26.7	38.4	34.9–42.0		1.47	1.23–1.74	1.34	1.14–1.57
Southeast	41.2	26.9	22.8–31.5		1.03	0.83–1.28	0.99	0.81–1.20
South	13.8	26.2	22.5–30.2		1	-	1	-
Midwest	8.4	29.7	25.7–34.0		1.13	0.92–1.39	1.04	0.86–1.26
Health Insurance				< 0.0001				
Yes	19.3	37.9	33.0–43.0		1.31	1.13–1.51	1.28	1.11–1.47
No	80.7	28.9	26.5–31.05		1	-	1	-
No. of emergency visits^e^				< 0.0001				
None	84.1	24.8	22.7–27.0		1	-	1	-
Two	9.5	51.5	44.3-58.7		2.08	1.76–2.45	1.79	1.52–2.11
Two or more	6.4	75.9	70.6-80.4		3.06	2.75–3.41	2.48	2.18–2.81
No. of hospitalizations^e^				< 0.0001				
None	95.1	29.0	26.7-31.5		1	-	1	-
One	4.0	59.8	50.6-68.3		2.06	1.75–2.43	1.31	1.10–1.56
Two or more	0.9	85.4	74.6-92.1		2.94	2.58–3.34	1.37	1.19–1.58

^a^ Percentages weighted by the sampling weights.

^b^ Pearson’s Chi-Squared test.

^c^ Poisson Regression.

^d^ 2013 Brazilian Economic Classification according to ABEP.

^e^ Regarding the 12 month-period prior to the interview.

The questionnaires were answered by the child’s mother in 76.1% of cases, followed by grandparent (9.6%) and father (8.6%).

Prevalence of chronic diseases was 9.9% (95%CI 8.6–11.3). Diabetes was reported by 0.4% of the sample (95%CI 0.2–0.8%) and chronic lung diseases by 5.2% (95%CI 4.3–6.4). Prevalence of acute conditions treated in the 15 days prior to the interview was 27.4% (95%CI 25.2–29.8). The most common were fever (8.4%; 95%CI 7.4–9.5), cold or flu (7.3%; 95%CI 6.2–8.5), pain (5.8%; 95%CI 4.8–7.0%) and infection (4.6%; 95%CI 3.8–5.5). The use of vitamins or mineral supplements was reported by 6.1% (95%CI 5.3–7.1).

Table 1 shows the prevalence of overall use of drugs and other products for therapeutic purposes (30.7%). Use was highest among children aged five or under, resident in the Northeast Region, with health insurance and who had used health services in the previous 12 months (one or more emergency visits and one or more hospitalizations). In the final model, these factors remained positively associated with the outcome, with statistical significance.

Table 2 shows the prevalence of drugs used to treat chronic (5.6%) and acute diseases (27.1%). Two years old or over, resident in the Southeast or South Regions, one or more emergency visits, and one or more hospitalizations remained positively associated with the use of medicines for chronic diseases. The positive association between health insurance and the outcome failed to show any statistical significance when adjusted for other variables.

**Table 2:** Prevalence of medicine use for chronic diseases and acute conditions, according to demographic, socioeconomic and medical variablesa. (N = 7,528)

Variable	Medicine use for chronic diseases				Medicine use for acute conditions			
	
	Prevalence (95%CI)	p^b^	PR_crude_ (95%CI)^c^	PR_adjusted_ (95%CI)^c^	Prevalence (95%CI)	p^b^	PR_crude_ (95%CI)^c^	PR_adjusted_ (95%CI)^c^
Overall	5.6 (4.7–6.7)				27.1 (24.8–29.4)			
Gender		0.174				0.302		
Female	4.9 (3.7–6.4)		1	1	26.1 (23.5–28.9)		1	1
Male	6.4 (4.9–8.3)		1.32 (0.88–1.98)	1.33 (0.91–1.94)	28.0 (25.0–31.3)		1.07 (0.94–1.23)	1.07 (0.94–1.23)
Age (years)		0.003				< 0.001		
< 2	3.7 (2.8–4.8)		1	1	46.5 (43.3–49.7)		2.32 (2.02–2.67)	2.09 (1.83–2.38)
2-5	7.5 (6.0–9.3)		2.05 (1.51–2.80)	2.14 (1.56–2.94)	32.9 (30.0–35.8)		1.64 (1.43–1.89)	1.44 (1.25–1.66)
6-12	5.2 (4.1–6.7)		1.43 (1.02–2.01)	1.88 (1.35–2.61)	20.0 (17.4–22.9)		1	1
Skin color		0.104				0.779		
White	6.4 (5.1–8.1)		1.30 (0.95–1.78)	1.10 (0.76–1.60)	27.0 (24.2–29.9)		0.98 (0.87–1.11)	0.98 (0.87–1.11)
Non-white	5.0 (3.9–6.3)		1	1	27.5 (24.7–30.4)		1	
Economic classification^d^		0.537				0.097		
A/B	5.6 (3.7–8.4)		1.18 (0.68–2.06)	1.18 (0.68–2.06)	22.7 (18.9–27.0)		0.80 (0.65–0.98)	0.87 (0.70–1.08)
C	6.0 (4.8–7.5)		1.26 (0.81–1.97)	1.26 (0.81–1.97)	27.6 (24.6–30.6)		0.97 (0.83–1.13)	1.05 (0.91–1.20)
D/E	4.8 (3.3–6.9)		1	1	28.5 (24.8–32.5)		1	1
Brazilian Region		< 0.001				< 0.001		
North	2.6 (1.6–4.0)		1	1	31.0 (24.3–38.6)		1.53 (1.14–2.06)	1.52 (1.19–1.96)
Northeast	4.3 (3.1–6.0)		1.69 (0.97–2.97)	1.56 (0.92–2.63)	35.5 (32.2–39.0)		1.76 (1.43–2.17)	1.61 (1.33–1.95)
Southeast	6.8 (5.0–9.2)		2.66 (1.54–4.61)	2.93 (1.76–4.88)	22.8 (19.1–27.0)		1.13 (0.87–1.46)	1.10 (0.88–1.38)
South	7.7 (6.3–9.5)		3.02 (1.83–4.98)	3.56 (2.21–5.73)	20.2 (16.7–24.2)		1	1
Midwest	4.4 (2.6–6.0)		1.55 (0.83–2.87)	1.45 (0.83–2.53)	27.5 (23.5–32.0)		1.36 (1.07–1.73)	1.26 (1.01–1.57)
Health Insurance		0.021				0.007		
Yes	8.4 (5.6–12.2)		1.68 (1.08–2.61)	1.28 (0.86–1.91)	32.2 (27.9–36.9)		1.25 (1.07–1.46)	1.26 (1.08–1.48)
No	5.0 (4.1–6.0)		1	1	25.8 (23.4–28.3)		1	1
No. of emergency visits^e^		< 0.001				< 0.001		
None	4.0 (3.3–5.0)		1	1	22.2 (20.1–24.3)		1	1
Two	7.5 (4.9–11.3)		1.86 (1.17–2.94)	1.55 (0.89–2.68)	45.2 (38.5–52.1)		2.04 (1.71–2.44)	1.80 (1.53–2.12)
Two or more	22.2 (16.2–29.7)		5.51 (3.82–7.93)	4.39 (2.62–7.34)	64.2 (58.0–70.1)		2.90 (2.56–3.27)	2.42 (2.12–2.78)
No. of hospitalizations^e^		< 0.001				< 0.001		
None	4.7 (3.8–5.7)		1	1	26.1 (23.9–28.5)		1	1
One	17.8 (11.3–26.9)		3.81 (2.30–6.28)	2.84 (1.50–5.38)	46.7 (38.0–55.7)		1.79 (1.45–2.20)	1.12 (0.92–1.36)
Two or more	44.3 (27.8–62.1)		9.46 (6.13–14.61)	3.84 (2.29–6.43)	55.9 (37.8–72.6)		2.14 (1.54–2.97)	0.98 (0.70–1.37)

^a^ Percentages weighted by the sampling weights.

^b^ Pearson’s Chi-Squared test.

^c^ Poisson Regression.

^d^ 2013 Brazilian Economic Classification according to ABEP.

^e^ Regarding the 12 month-period prior to the interview.

The following factors were associated with the use of medicines to treat acute conditions in the crude analysis: five years old or younger, resident in the North, Northeast, and Midwest Regions, health insurance, and use of health services. In the multivariate analysis, these factors remained positively associated with the outcome and statistically significant, except for the number of hospitalizations. Belonging to economic classes A/B was negatively associated with the outcome, but not sustained after adjustment for covariates.

Of the total number of products or drugs reported (5,185), 91.4% (95%CI 89.9–92.6) corresponded to medicines, 1.7% (95%CI 1.3–2.4) to dietary supplements (low-dose vitamin combinations), 1.2% (95%CI 0.9–1.7) to herbal medicines, 1.1% (95%CI 0.7–1.9) to plants or teas, 0.5% (95%CI 0.3–0.8) to food (honey compounds), and 0.1% (95%CI 0.0–0.7) to homeopathic medicines. The name of 3.9% (95%CI 3.0–5.1) of the products was not identified.

Table 3 shows the prevalence of medicines most used by Brazilian children (equal to or above 0.5%). Considering the total sample, the highest prevalence in drug use was observed for dipyrone (5.1%; 95%CI 4.3–6.1), paracetamol (4.3%; 95%CI 3.6–5.1), and amoxicillin (2.7, 95%CI 2.2–3.4).

**Table 3:** Prevalence of most often used medicines (prevalence of use ≥ 0.5%) by children, by age groupa. (N = 7,528)

Drug^b^	< 2 years (n = 2,302)		2-5 years (n = 3,671)		6-12 years (n = 1,555)		Total (n = 7,528)	
			
	Prevalence	95%CI	Prevalence	95%CI	Prevalence	95%CI	Prevalence	95%CI
Dypirone	5.7	4.5–7.2	6.2	4.9–7.7	4.6	3.4–6.1	5.1	4.3–6.1
Paracetamol	10.1	8.6–11.8	5.9	4.6–7.5	2.2	1.5–3.2	4.3	3.6–5.1
Amoxicilin	3.4	2.6–4.6	4.0	3.2–5.0	2.0	1.3–3.1	2.7	2.2–3.4
Ibuprofen	3.9	3.0–5.0	3.3	2.5–4.5	1.7	1.0–2.8	2.4	1.9–3.1
Ascorbic acid	6.9	5.5–8.6	1.9	1.3–2.9	0.3	0.1–0.7	1.6	1.3–2.0
Dexchlorpheniramine	1.5	1.0–2.2	1.7	1.2–2.4	1.6	0.9–2.5	1.6	1.2–2.2
Ambroxol	1.3	0.8–2.0	1.3	0.8–2.3	0.8	0.4–1.4	1.0	0.7–1.4
Prednisolone	1.7	1.1–2.8	1.6	1.2–2.2	0.6	0.3–1.3	1.0	0.7–1.4
Ferrous sulphate	4.0	3.0–5.5	0.4	0.2–0.7	0.4	0.1–1.1	0.9	0.6–1.3
Cholecalciferol; retinol	5.7	3.9–8.3	0.2	0.1–0.6	0	0.0–0.0	0.8	0.6–1.2
Multivitamins	4.0	3.0–5.3	0.4	0.2–0.6	0.1	0.0–0.3	0.7	0.5–0.9
Dimethicone	3.7	2.8–5.1	0.6	0.2–1.8	0.1	0.0–0.6	0.7	0.5–1.1
Loratadine	0.6	0.3–1.1	0.9	0.6–1.4	0.6	0.3–1.2	0.6	0.4–1.0
Fluticasone	0	0.0–0.3	0.7	0.2–1.8	0.7	0.3–2.0	0.6	0.2–1.6
Sulfamethoxazol; trimethropim	0.9	0.6–1.4	1.0	0.7–1.5	0.2	0.0–0.6	0.5	0.3–0.7
Salbutamol	0.7	0.4–1.4	0.9	0.6–1.3	0.3	0.1–0.9	0.5	0.3–0.8
Nimesulide	0.4	0.2–0.8	0.5	0.3–0.9	0.5	0.2–1.0	0.5	0.3–0.8
Cephalexin	0.9	0.6–1.5	0.8	0.4–1.5	0.2	0.1–0.7	0.5	0.3–0.8
Amoxicilin; potassium clavulanate	0.4	0.2–1.1	0.9	0.4–2.0	0.2	0.1–1.0	0.5	0.2–0.9
Phenoterol	0.4	0.2–1.0	0.7	0.3–1.4	0.4	0.2–1.1	0.5	0.3–0.9

^a^ Percentages weighted by the sampling weights.

^b^ Excluding supplements, food, plants and herbal medicines.

Tables 4 and 5 show, for each age group, the drugs reported in treating chronic and acute diseases, respectively. Prominent among the total number of drugs to treat chronic diseases were those acting on the respiratory system (salbutamol, dexchlorpheniramine, fluticasone, and phenoterol), in all age groups (Table 4). Painkillers were the most cited drugs for treating acute conditions (Table 5).

**Table 4:** Most used medicines (frequency > 1.0%) for chronic diseases, by age groupa.

< 2 years			Preschool age (2-5 years)			School age (6-12 years)		
		
(n = 119 medicines)	%	95%CI	(n = 295 medicines)	%	95%CI	(n = 115 medicines)	%	95%CI
Salbutamol	8.4	3.5–18.7	Salbutamol	5.8	3.4–9.9	Carbamazepine	9.1	3.8–20.4
Prednisolone	7.2	3.0–15.9	Phenoterol	5.3	2.6–10.6	Fluticasone	9.1	2.8–26.0
Dexchlorpheniramine	7.0	3.0–15.4	Fluticasone	5.0	1.7–13.4	Dexchlorpheniramine	5.6	2.0–15.0
Amoxicilin	6.6	2.7–15.4	Prednisolone	4.5	2.5–8.1	Phenoterol	4.9	1.8–12.4
Beclomethasone	5.5	1.5–18.0	Ipratropium	4.2	1.7–9.7	Salbutamol	4.7	2.0–10.7
Ferrous sulphate	4.1	1.2–13.0	Phenobarbital	4.0	1.5–9.9	Budesonide; formoterol	4.6	1.4–14.0
Phenobarbital	3.7	1.5–8.9	Sodium chloride	3.7	0.9–13.9	Ipratropium	3.5	0.9–12.5
Bethametasone; Dexchlorpheniramine	3.2	1.1–9.0	Beclomethasone	3.6	1.7–7.4	Levothyiroxine	3.1	0.7–12.8
Loratadine	3.1	0.7–12.7	Risperidone	3.4	0.7–14.4	Methylphenidate	3.0	0.6–12.8
Sodium chloride	2.6	0.9–7.9	Desloratadine	3.4	0.7–14.3	Loratadine	3.0	0.8–10.4
Prednisone	2.6	0.3–17.4	Ketotifen	2.9	0.9–9.3	Budesonide	3.0	1.0–8.2
Bronpheniramine; Phenylephrine	2.5	0.5–11.9	Ascorbic acid	2.9	0.4–17.4	Prednisolone	2.9	1.0–7.8
Multivitamins	2.4	0.3–16.0	Vitamins B Complex^b^	2.9	0.4–17.4	Tacrolimus	2.5	0.5–12.9
Dexamethasone	2.3	0.7–7.6	Pericyazine	2.9	0.5–15.5	Human insulin	2.5	0.3–16.6
Domperidone	2.2	0.7–6.8	Dexchlorpheniramine	2.3	1.1–5.0	Methlylprednisolon acetate	2.3	0.3–13.5
Ketotifen	2.2	0.3–14.1	Carbamazepine	2.2	0.6–7.2	Divalproex sodium	2.1	0.3–14.0
Cromoglycate	2.2	0.3–13.0	Budesonide	2.1	0.9–4.6	Oxcarbazepine	2.1	0.3–13.5
Acebrophylline	2.1	0.7–6.0	Montelukast	2.0	0.8–4.9	Topiramate	2.1	0.3–13.5
Ambroxol	1.9	0.4–9.3	Betamethasone; Dexchlorpheniramine	2.0	0.6–5.9	Folic acid	1.7	0.4–7.8
Folic acid	1.9	0.3–12.9	Amoxicilin	1.9	0.8–4.3	Olopatadine	1.5	0.2–10.9
Ibuprofen	1.8	0.6–4.9	Dihidroxizine	1.8	0.3–10.4	Phenobarbital	1.4	0.2–7.8
Thiabendazol	1.6	0.2–11.3	Loratadine	1.7	0.6–4.4	Calcitriol	1.2	0.2–8.7
Phenoterol	1.5	0.2–10.6	Clobutinol; doxylamine	1.7	0.2–10.8	Biperiden	1.2	0.2–8.3
Desloratadine	1.3	0.3–6.1	Calcium; nicotinamide; panthenol; abscorbic acid	1.6	0.2–10.9	Chlorpromazine	1.2	0.2–8.3
Amoxicilin; potassium Clavulanate	1.3	0.2–9.1	Ibuprofen	1.4	0.5–4.2	Dimethicone	1.2	0.2–8.3
Ferripolimaltose	1.2	0.2–6.1	Acebrophylline	1.4	0.5–3.9	Dipyrona; scopolamine	1.2	0.2–8.3
Beclomethasone; salbutamol	1.1	0.1–7.5	Mometasone	1.3	0.5–3.4	Haloperidol	1.2	0.2–8.3
Budesonide	1.1	0.2–6.8				Omeprazole	1.2	0.2–8.3
Cholecalciferol; retinol	1.1	0.2–6.8				Sodium picosulfate	1.2	0.2–8.7
Ranitidine	1.1	0.2–6.8						

^a^ Excluding herbal medicines and other products for therapeutic purpose reported by respondents (dietary supplements, food, plants and homeopathic products).

^b^ Biotin, cyanocobalamin, pyridoxine, thiamine, riboflavin, nicotinamide, panthenol and calcium pantothenate.

**Table 5:** Most used medicines (frequency > 1.0%) for acute conditions, by age groupa.

< 2 years			Preschool age (2-5 years)			School age (6-12 years)		
		
(n = 1,829 medicines)	%	95%CI	(n = 1,868 medicines)	%	95%CI	(n = 385 medicines)	%	95%CI
Paracetamol	13.1	11.4–15.1	Dipirone	13.3	10.7–16.3	Dipirone	18.5	14.3–23.6
Ascorbic acid	8.9	7.1–11.0	Paracetamol	12.8	10.3–15.8	Paracetamol	9.1	6.2–13.0
Dipyrone	7.3	5.9–9.1	Amoxicilin	8.3	6.7–10.3	Amoxicilin	8.0	5.2–12.0
Cholecalciferol; retinol	7.1	4.9–10.1	Ibuprofen	6.9	5.2–9.2	Ibuprofen	7.1	4.5–11.2
Ibuprofen	5.0	3.9–6.4	Ascorbic acid	3.4	2.5–4.6	Dexchlorpheniramine	4.8	2.9–7.9
Multivitaminas	5.0	3.7–6.6	Dexchlorpheniramine	3.2	2.2–4.5	Ambroxol	3.1	1.7–5.6
Ferrous sulphate	4.9	3.6–6.7	Ambroxol	2.8	1.6–4.8	Nimesulide	2.0	1.0–4.1
Dimethicone	4.8	3.6–6.4	Prednisolone	2.4	1.7–3.5	Mebendazole	1.7	0.6–4.5
Amoxicilin	4.1	3.1–5.5	Sulfamethoxazole; trimethoprim	2.1	1.4–3.3	Prednisolone	1.6	0.5–4.5
Ferripolimaltose	2.0	1.3–3.2	Amoxicilin; potassium clavulanate	1.8	0.8–4.2	Ferrous sulphate	1.6	0.5–4.6
Bronpheniramine; phenylephrine	1.9	1.2–3.0	Cephalexine	1.6	0.8–3.0	Albendazole	1.4	0.4–4.6
Prednisolone	1.8	1.1–2.9	Azitromycin	1.5	0.5–4.0	Fexofenadine	1.3	0.4–4.6
Ambroxol	1.5	1.0–2.4	Loratadine	1.4	0.9–2.3	Loratadine	1.3	0.5–3.0
Dexchlorpheniramine	1.5	1.0–2.2	Dimethicone	1.3	0.4–3.9	Azithromycin	1.1	0.3–3.8
Soidum chloride	1.2	0.7–2.2	Bromopride	1.3	0.5–3.2			
Sulfamethoxazole; trimethoprim	1.2	0.8–1.8	Nimesulide	1.1	0.7–1.8			
Cephalexina	1.1	0.6–1.9	Vitamins B complex^b^	1.1	0.4–2.7			
			Betamethasone; dexchlorpheniramine	1.1	0.6-2.0			

^a^ Excluding herbal medicines and other products for therapeutic purpose reported by respondents (dietary supplements, food, plants and homeopathic products).

^b^ Cyproheptadine, ascorbic acid, propylene glycol, thiamine, pyridoxine, nicotinamide and riboflavin.

## DISCUSSION

This is the first study of drug use in children with a representative sample of the Brazilian urban population, providing estimates of the prevalence of drug use based on the chronic or acute nature of the treated health problem. The profile of medicine users and the most frequently used pharmacological agents in each phase of childhood are also described in this study.

Prevalence of overall drug use was lower than that observed in studies involving drug use in the previous seven days by children under 12 living in the US (56.0%)^25^ and individuals aged zero to 17 in Germany (50.8%)^15^. However, it was similar to that observed in surveys based on two-week periods in Spain^4^ and Norway^10^. In Spain, surveys with children aged zero to 15 carried out in 1993 and 2003 showed prevalence rates of 36.8% and 34.0%, respectively^4^. In Norway, about 25.0% of children up to four years of age used medicines, decreasing to approximately 20.0% among older children^10^. Compared to studies carried out in Brazil, prevalence was lower than that observed in Salvador, BA^23^, Bagé, RS^19^, and the Jequitinhonha Valley, MG^8^.

The significant difference between prevalence of drug use to treat chronic diseases and acute conditions was expected, considering that this population group is characterized by predominantly healthy individuals. Although Cox et al.^7^ (2008) observed an increase in the prevalence of certain chronic diseases among American children aged five to 19, children are most commonly affected by acute medical conditions, directly influencing how drugs are used (continuous *versus* occasional use).

The lack of difference in prevalence of overall drug use or related to acute events between boys and girls is in accordance with recent studies^4^ ^,^ ^8^ ^,^ ^19^. The decrease in the prevalence of overall drug use or related to acute conditions as age increases corroborates findings from studies that showed increased use among children under two years old, compared to older children^4^ ^,^ ^12^ ^,^ ^15^ ^,^ ^16^. These findings indicate that the first 24 months of life, when children are more susceptible to diseases and receive more medical care, either elective or emergency, are followed by a period of low incidence of acute conditions, which lasts approximately until early adolescence. Menarche-related events entail the use of painkillers and contraceptives by adolescent girls, contributing to increased prevalence in this age group^2^.

The data of this study show that the profile of drug use to treat acute or chronic health conditions differs according to region. The highest prevalence of use for chronic conditions was observed in the South and Southeast Regions, associated with the higher prevalence of chronic respiratory diseases in these regions (data not shown). The use of drugs for acute conditions, higher in the Northeast and North Regions, can be explained by the higher frequency of medicine use for diarrhea and other gastrointestinal disorders, and the higher percentage of self-medication in these regions, according to PNAUM data not shown.

An overall positive association was found between use of health services and use of medicines, in line with data from the 2003 Spanish survey^4^. Contact with health care services provides greater chances of children receiving medical prescriptions, which may lead to the routine use of prescription drugs or new posterior use.

Medicines to control pain and fever are among the most used, according to previous studies^4^ ^,^ ^8^ ^,^ ^19^ ^,^ ^25^. Dipyrone is the most used analgesic and antipyretic, followed by paracetamol and ibuprofen. This pattern differs from that observed in studies carried out in countries where the use of dipyrone has been banned or restricted, as in the US, where ibuprofen stands out as the second most used painkiller^25^. Amoxicillin was the most used antimicrobial in all age groups, with higher prevalence among children aged two to five (4.0%) and lower prevalence among children aged six to 12 (2.0%). In the US, amoxicillin also ranked as the most widely used antimicrobial, with higher prevalence among children under two years of age (5.1%) and lower prevalence in the six to 11 age group (1.5%)^25^. With the exception of dipyrone, whose safety controversies have persisted for years, the other drugs mentioned are considered effective and safe, with good evidence for pediatric use^21^.

Another fact that draws attention relates to the use of vitamins, alone or in combination. The use of ascorbic acid alone was superior to the use of multivitamins in all age groups investigated, in contrast to the findings of a US study, in which multivitamins were mentioned more often^25^ (2009). In Salvador, vitamin C was used more often than multivitamins, although the difference observed in prevalence of use was smaller^24^.

Prominent among the 10 most mentioned drugs for use in chronic diseases are those indicated for respiratory diseases such as asthma and rhinitis, followed by those with therapeutic use in epilepsy. Among children below two years old it is observed use of various drugs without FDA approval for this age group and no indication in the standard package inserts approved by ANVISA^d^. Among the most frequently cited drugs with restrictions for use in this age group are salbutamol, dexchlorpheniramine (alone or combined with betamethasone), loratadine, and brompheniramine + phenylephrine. It should be noted that, in some cases, drugs not approved by the FDA are indicated in Brazilian standard package inserts, as is the case of betamethasone and ambroxol^d^ ^,^ ^e^. If, on the one hand, the use of non-approved drugs may represent a potentially inappropriate administration of drugs whose efficacy and safety have not been established, on the other it may reflect the lack of therapeutic options to treat health conditions prevalent in children, such as asthma, rhinitis, and acute or chronic coughing, among others^5^.

Among children under two years of age, one of the most used drugs to treat chronic diseases was amoxicillin. This can be explained by recurrent respiratory infections in this age group, identified as chronic by parents or caregivers for occurring with high frequency in short periods of time. This finding should be considered in the context of the increase in antimicrobial resistance rates reported worldwide and in Brazil^11^ ^,^ ^22^.

Regarding medicines most used in acute conditions, important differences are found in the characteristics of the most commonly used drugs in the first two years of life compared to older children. The presence of ascorbic acid, cholecalciferol+retinol, ferrous sulfate and multivitamins among the 10 drugs most used by children under 12 shows that supplementation with vitamins alone or in combination in the first years of life is common practice in pediatrics, despite not always being supported by evidence^9^ ^,^ ^14^ ^,^ ^26^. Among preschool children, what draws attention is the higher frequency of antimicrobials, including amoxicillin, sulfamethoxazole+trimethropim, and amoxicillin+clavulanate, among the 10 most cited drugs. These results suggest that with increasing age, the higher incidence of infectious diseases, particularly respiratory, leads to greater use of antimicrobials. The age groups of two to five years and six to 12 years show the use of nimesulide, a drug with risk of liver toxicity and Reye’s syndrome in children with symptoms of viral infection, being contraindicated for children under the age of 12 according to the standard package insert approved by ANVISA.

This study has limitations. It uses information provided by interviewees on the presence of chronic disease, which can lead to overestimation of prevalence of use. On the other hand, socio-cultural aspects involving the use of medicines, particularly when related to risk groups, may contribute to parents (the main respondents in this research) giving socially desirable answers^4^ ^,^ ^25^. Thus, omissions in reporting the use of certain medicines cannot be ruled out. Finally, the use of drugs for diseases that are typical in colder months, especially in the South and Southeast Regions, may be underrepresented, given that data collection occurred during spring and summer.

The results of this study indicate that a considerable percentage of Brazilian children make use of medicines, especially to treat acute conditions. Children using drugs for chronic diseases have a different demographic profile compared to those using drugs for acute conditions according to gender, age, and geographic region. Among the most commonly used drugs are medicines acknowledged as effective for pediatric use and others whose effectiveness and safety are uncertain when used in children, particularly those under the age of two.

## Footnotes

a Associação Brasileira de Empresas de Pesquisa. Critério de Classificação Econômica Brasil 2013. São Paulo (SP): ABEP; 2013 [cited 2016 Feb 29]. Available from: http://www.abep.org/criterio-brasil

b Food and Drug Administration. Guidance for industry:E11 clinical investigation of medicinal products in the pediatric population. Rockville (MD); 2000 [cited 2016 Feb 29]. Available from: http://www.fda.gov/downloads/Drugs/GuidanceCompliance RegulatoryInformation/Guidances/UCM073143.pdf

c Instituto Brasileiro de Geografia e Estatística. Censo demográfico 2010: características da população e dos domicílios: resultado do universo. Rio de Janeiro (RJ): IBGE; 2011 [cited 2016 Feb 29]. Available from: http://www.ibge.gov.br/home/estatistica/populacao/censo2010/caracteristicas_da_populacao/default_caracteristicas_da_populacao.shtm

d Ministério da Saúde, Agência Nacional de Vigilância Sanitária. Bulário eletrônico. Brasília (DF); s.d [cited 2016 Feb 20]. Available from: http://www.anvisa.gov.br/datavisa/fila_bula/index.asp

e Klasco RK, editor. DRUGDEX® System. Greenwood Village (CO): Thomson MICROMEDEX; 2016.
